# The Hitchhiker’s Guide to Neurophenomenology – The Case of Studying Self Boundaries With Meditators

**DOI:** 10.3389/fpsyg.2020.01680

**Published:** 2020-07-21

**Authors:** Aviva Berkovich-Ohana, Yair Dor-Ziderman, Fynn-Mathis Trautwein, Yoav Schweitzer, Ohad Nave, Stephen Fulder, Yochai Ataria

**Affiliations:** ^1^Department of Learning, Instruction and Teacher Education, Faculty of Education, University of Haifa, Haifa, Israel; ^2^Department of Counseling and Human Development, Faculty of Education, University of Haifa, Haifa, Israel; ^3^Edmond J. Safra Brain Research Center, University of Haifa, Haifa, Israel; ^4^The Integrated Brain and Behavior Research Center (IBBRC), University of Haifa, Haifa, Israel; ^5^Department of Psychosomatic Medicine and Psychotherapy, Medical Center – University of Freiburg, Freiburg im Breisgau, Germany; ^6^Department of Cognitive Sciences, The Hebrew University of Jerusalem, Jerusalem, Israel; ^7^The Israel Insight Society (Tovana), Karmiel, Israel; ^8^Department of Psychology, Tel-Hai Academic College, Tel Hai, Israel

**Keywords:** neurophenomenology, sense of self, embodied self, self boundaries, meditation, magnetoencephalography (MEG)

## Abstract

This paper is a practical guide to neurophenomenology. Varela’s neurophenomenological research program (NRP) aspires to bridge the gap between, and integrate, first-person (1P) and third-person (3P) approaches to understanding the mind. It does so by suggesting a methodological framework allowing these two irreducible phenomenal domains to relate and reciprocally support the investigation of one another. While highly appealing theoretically, neurophenomenology invites researchers to a challenging methodological endeavor. Based on our experience with empirical neurophenomenological implementation, we offer practical clarifications and insights learnt along the way. In the first part of the paper, we outline the theoretical principles of the NRP and briefly present the field of 1P research. We speak to the importance of phenomenological training and outline the utility of cooperating with meditators as skilled participants. We suggest that 1P accounts of subjective experience can be placed on a complexity continuum ranging between thick and thin phenomenology, highlighting the tension and trade-off inherent to the neurophenomenological attempt to naturalize phenomenology. We then outline a typology of bridges, which create mutual constraints between 1P and 3P approaches, and argue for the utility of alternating between the bridges depending on the available experimental resources, domain of interest and level of sought articulation. In the second part of the paper, we demonstrate how the theory can be put into practice by describing a decade of neurophenomenological studies investigating the sense of self with increasing focus on its embodied, and minimal, aspects. These aspects are accessed via the dissolution of the sense-of-boundaries, shedding new light on the multi-dimensionality and flexibility of embodied selfhood. We emphasize the evolving neurophenomenological dialogue, showing how consecutive studies, placed differently on the thin-to-thick 1P continuum, advance the research project by using the bridging principles appropriate for each stage.

## Introduction

Toward the end of the last millennium, Francisco Varela, the Chilean biologist, philosopher, and neuroscientist, put forth an ambitious proposal, the neurophenomenological research program (NRP). The NRP attempts at paving a methodological path for bridging the ‘explanatory gap’ in our understanding of how to integrate first-person (1P) phenomenological and third-person (3P) physiological features of the mind. Instead of ineffective attempts to close the conceptual gap between subjective experience and physical matter, also referred to as the ‘Hard Problem of Consciousness’ (Chalmers, 1995), the NRP suggests reframing the gap. To this end, it defines a methodological strategy for integrating phenomenological and neurobiological accounts under one research program, or in Varela’s words, for creating “meaningful bridges between two irreducible phenomenal domains” (Varela, 1996, p. 330). The NRP has been described as “a quest to marry modern cognitive science and a disciplined approach to human experience” (Ibid. p. 340) and it is this harmonious relationship which we pursue.

With more than two decades since its introduction, numerous theoretical studies have illuminated the importance, ingenuity and potential of the NRP, yet not as many have succeeded in actually implementing it as an empirical methodology (for a short review see Tables 1, 2; for other reviews, see Lutz and Thompson, 2003; Bayne, 2004; Thompson et al., 2005; Lutz, 2006, 2007; Bitbol, 2012; Bockelman et al., 2013; Ataria, 2017; Berkovich-Ohana, 2017). Clearly, NRP’s strong philosophical appeal is merely the grounds from which a scientific paradigm may grow and mature to address the highly ambitious and demanding challenge at stake – “an open-ended quest for resonant passages between human experience and cognitive science” (Varela, 1996, p. 346). If the challenge is to be met on Varela’s terms, it can only be done based on rigorous empirical practice. It is the goal of this paper to support those who wish to embark on this journey and implement the NRP by highlighting some of the important lessons learnt in neurophenomenological studies done in recent years, including our own.

**TABLE 1:** A list of papers relating to neurophenomenology (NP) on the theoretical level.

Theoretical perspectives	Papers
What is NP	Varela, 1996; Lutz, 2002, 2007; Lutz and Thompson, 2003; Rudrauf et al., 2003; Thompson, 2006
’Front-loading’ phenomenology into experimental design	Gallagher and Sørensen, 2006
Neurofeedback useful for NP	Bagdasaryan and Le Van Quyen, 2013
Hypnosis useful for NP	Lifshitz et al., 2013
Astronaut simulation and challenges of NP	Bockelman et al., 2013
Dreams and challenges of NP	Solomonova et al., 2014
Kantian perspective on NP	Khachouf et al., 2013
NP in the context of in dyadic movement	Stuart, 2013
Application of NP in affective neuroscience	Colombetti, 2013
NP usefulness in psychology	Gordon, 2013
NP usefulness in ECoG	Petitmengin and Lachaux, 2013
NP justification in practice	Strle, 2013
Philosophical attack and justification	Bitbol and Antonova, 2016; Kirchhoff and Hutto, 2016
Advantage of training scientists in contemplation	Desbordes and Negi, 2013
NP applied to time consciousness	Varela, 1999
NP usefulness in pain	Price et al., 2002
Combining descriptive experience sampling with 3P	Hurlburt et al., 2017
Combining 2P methods	Froese et al., 2011; Olivares et al., 2015
Philosophical roots of NP and enaction	Vörös et al., 2016
An externalist extension of NP	Beaton, 2013
Adding a cardiac-affective dimension to NP	Depraz and Desmidt, 2018
Distinguishing mild vs. radical NP	Petitmengin, 2017
The feasibility of NP	Ataria, 2017
Neurofeedback useful for NP	Bielas and Michalczyk, 2017
NP usefulness to understand trauma	Ataria et al., 2019
Application of NP to microdreams	Nielsen, 2017
Phenomenological matrix of mindfulness	Lutz et al., 2015
Phenomenologically constrained neurocomputational model of the self	Williford et al., 2018
Brain dynamics from the perspective of NP	Fazelpour and Thompson, 2015
NP of surprise	Bitbol,2019b
Neuroscience and inner experience	Price and Barrell, 2012

**TABLE 2:** A list of papers employing neurophenomenological empirical paradigms.

1P/2P	3P	Papers
Attentional state	EEG	Lutz et al., 2002
	Behavior	Van den Bussche et al., 2013; Zanesco et al., 2013
Epilepsy	EEG	Le Van Quyen and Petitmengin, 2002; Petitmengin et al., 2006, 2007
Sense-of-self (time/space/boundary/identification)	MEG	Berkovich-Ohana et al., 2013a; Dor-Ziderman et al., 2013; Ataria et al., 2015; Dor-Ziderman et al., 2016
	fMRI	Garrison et al., 2013
Hypnosis	EEG	Cardeña et al., 2013
Astronaut simulation	EEG/fNIRS	Reinerman-Jones et al., 2013
Mind-wandering	fMRI	Christoff et al., 2009; Allen et al., 2013
Emotion	fMRI	Northoff and Heinzel, 2006
Yoga/attention/emotion	HRV	Mackenzie et al., 2014
Neuro inspires pheno		De Haan et al., 2013; Valenzuela Moguillansky et al., 2013
Resting state	fMRI/EEG	Diaz et al., 2013
Intention to act	EEG	Guggisberg and Mottaz, 2013; Jo et al., 2014, 2015
Dreaming	PET/fMRI	Fox et al., 2013
Memory	HRV/GSR	McCall et al., 2015
Pain under hypnosis	PET	Rainville et al., 1997
Language/auditory processing	fMRI	Kühn et al., 2014; Hurlburt et al., 2016
Descriptive experience sampling	Verbal behavior	Hurlburt et al., 2002
Approach-avoidance	Behavior	Baquedano and Fabar, 2017
Surprise in depression	ECG	Depraz et al., 2017
Meditative state	Behavior	Abdoun et al., 2019
Psychedelic state	EEG	Timmermann et al., 2019

**EEG, electroencephalography; MEG, magnetoencephalography; fMRI, functional magnetic resonance imaging; HRV, heart rate variability; PET, positron emission tomography; GSR, galvanic skin response; fNIRS, functional near-infrared spectroscopy*.*

In the first part of the paper, we present Varela’s NRP while specifically focusing on various issues regarding the execution of an empirical phenomenological investigation. We point toward an inherent tension within the NRP concerning the challenge of naturalization in the face of the complexity and intricacy of 1P data. In this regard, we offer a diverse typology of bridges which exemplifies the concept of ‘mutual constraints,’ and argue for the need to gradually and interchangeably weave them through the developmental stages of an evolving research program.

The second part of this paper demonstrates our own decade-long implementation of the NRP focused on breaking new ground in the scientific understanding of self consciousness, with particular interest in alterations in the sense of embodied self and the minimal self in meditative experience. We found it helpful to repeatedly circulate between different forms of phenomenological inquiry and a variety of cognitive and neuroscientific tools, and argue that it is the ongoing development of a dialogue between these two perspectives that enables novel insights. We close the paper by presenting our current aims of advancing the understanding of self consciousness by employing an ongoing mature and pragmatic neurophenomenological study of the sense of self boundaries and their dissolution.

## Varela’s Neurophenomenological Research Project (NRP)

The Archimedean point of the NRP is acknowledging the irreducible nature of conscious experience: “Lived experience is where we start from and where all must link back to, like a guiding thread” (Varela, 1996, p. 334). Stemming from the phenomenological tradition (see Supplementary Appendix 1 for an outline of its historical roots), this notion has far-reaching implications for how we conceptualize nature and our place as embodied cognitive agents within it. It reminds us of the ineradicability of our own standpoint as humans (or cognitive scientists) and motivates a search for an understanding of the co-determination of mind and world as a middle way between the dead-ends of realism or idealism (Varela et al., 1991). Theoretically, this aim is pursued within the enactive approach, which is increasingly becoming a stronghold in cognitive science driving manifold research agendas (see Stewart et al., 2010; Vörös et al., 2016 and Newen et al., 2018 for overviews). Given their common theoretical starting point, neurophenomenology can be considered as a promising methodology of enactivism (Vörös et al., 2016). It seeks to provide a pragmatic methodological framework in which cognitive neuroscience can rigorously integrate a disciplined examination of conscious experience. This notion stresses the necessity of acquiring refined and reliable 1P descriptions in order to advance toward “a model that can account for both the phenomenology and neurobiology of consciousness in an integrated and coherent way” (Thompson et al., 2005, p. 87). The emphasis here is on forming constant circulation and dialogue between these two domains of inquiry which would allow an exploration of “the bridges, challenges, insights and contradictions between them” (Varela, 1996, p. 343).

Inspired by the phenomenological tradition, the call for a systematic exploration of lived experience put forward by the NRP has received considerable attention in recent years. In essence, such exploration is grounded in a set of practices that generally allow subjects to increase their sensitivity to their moment to moment experience (Varela and Shear, 1999; Petitmengin, 2011). Stemming from Edmund Husserl’s concepts of the phenomenological reduction and the epoché (Husserl, 1970; Bitbol, 2019a; for elaboration, see Supplementary Appendix 1), there are currently various first person (1P) and second person (2P)^1^ methods that promote a shift in attitude from the natural theory-laden absorption with the contents of one’s experience, to an awareness of the various affective, attentional and structural features of experience (discussed further in section “Investigating Lived Experience – From Thin to Thick Phenomenology”). In other words, instead of focusing on the ‘what,’ subjects are encouraged to bracket assumptions and presuppositions *about* their experience and become aware of the ‘how’ of experience, that is, the subjective mode of appearance and the dynamic intentional acts involved in the flow of experience (Petitmengin, 2006; Bitbol, 2019a). This dimension of experience is often termed by phenomenologists as pre-reflective awareness pointing toward the tacit, direct and non-inferential awareness of one’s experience as it is lived through, prior to any second-order reflection *on* experience (Nagel, 1974; Merleau-Ponty, 1996; Zahavi, 2002).

1P accounts have a vital role to play, along neuroscience and physiology, in beginning to bridge the explanatory gap *a la* Varela. The working hypothesis of the NRP is that “phenomenological accounts of the structure of experience and their counterparts in cognitive science relate to each other through reciprocal constraints” (Varela, 1996, p. 343). It is this type of circulation between the two perspectives which is at the focus of this paper and is described in more detail in Section “Building Bridges Between Phenomenology and Physiology – Mutual Constraints.” By reciprocal constraints Varela means both using neuroscientific accounts to illuminate previously unnoticed aspects of mental experience, and on the other hand, guiding the empirical questions, analysis and interpretation of neurobiological findings in light of the phenomenal invariants of the mental experience. 1P data generated from the phenomenology of mental processes “can provide additional, valid information about externally uncontrollable aspects of mental activity, and this information can be used to detect significant patterns of dynamic activity at the neural level” (Thompson et al., 2005, pp. 45–46). Thus on a methodological level, the NRP suggests explicitly and rigorously incorporating phenomenological investigation into experimental setup and design.

### Investigating Lived Experience – From Thin to Thick Phenomenology

The question of the importance, validity and place of the investigation of lived experience within science has seen many diverse conceptualizations throughout history. It is obviously beyond the scope of this paper to address the often-encountered notion (in cognitive neuroscience circles in particular) that subjective experience cannot be fully studied using the scientific method (but see Varela and Shear, 1999; Depraz et al., 2003; Froese et al., 2011; Olivares et al., 2015; Kordeš and Demšar, 2018). The aim of this section is to briefly present the state of the art in the developing field of 1P research, highlighting the demands, obstacles and practical implications imposed on neurophenomenological studies. We then present the concepts of ‘thin’ and ‘thick’ phenomenology as an organizational tool for the different levels of depth which specify 1P data in the context of the NRP.

As lived experience is a constantly changing, multi-layered and highly complex flux, its exploration is challenging. It requires employing a certain mental gesture of reflection toward one’s own experience that differs considerably from casual ‘introspection.’ Though formulated in various ways, it is often argued by 1P researchers that “becoming aware of lived experience is a skill that can and should be learned and practiced” (Froese et al., 2011, p. 254). Indeed, a rather demanding element in Varela’s radical vision is that cognitive science students interested in mental experience “must inescapably attain a level of mastery in phenomenological examination” through sustained training (Varela, 1996, p. 347). While such training may intuitively seem valuable to researchers’ own understanding of the phenomena they are investigating (Jack and Roepstorff, 2002; Gallagher and Zahavi, 2008), in the context of experimental neurophenomenological studies this effort is often centered on the rigorous acquisition of reliable 1P data from study participants. One suggested way to acquire such data is by using phenomenologically trained subjects (discussed in section “Cooperation With Meditators As Skilled Participants”), while another is based on interview techniques collectively known as second-person (2P) methods. Thus, first and second -person methods serve as supports for the examination of lived experience. 1P methods cultivate the capacity for sustained awareness, helping subjects gain access to aspects of their experience that are lived through but mostly remain unnoticed (Petitmengin, 2006; Froese et al., 2011); while the disciplined nature of 2P methodologies enables the systematic gathering of reliable phenomenological reports which can then be incorporated in various ways into neuroscientific research (elaborated in the following sections).

Recently, several interview techniques designed for detailed and careful description of subjective experience have been developed (for an overview, see Froese et al., 2011; Olivares et al., 2015). Mediated by the guidance of a skillful interviewer, they allow “a person, who may not even have been trained, to become aware of his or her subjective experience, and describe it with great precision” (Petitmengin, 2006, p. 334). Two notable 2P methods, Micro-phenomenology (Petitmengin, 2006; also known as ‘Elicitation Interview,’ Vermersch, 2009) and Descriptive Experience Sampling (‘DES,’ Hurlburt and Akhter, 2006), are based on retrospective examination of past experiences framed and guided by an empathetically tuned phenomenological investigator. In the context of neurophenomenology, these methods systematize the phenomenological research procedure, thus serving as valuable tools for performative coherence and scientific rigor. While the issue of validity of such procedures remains widely contentious (Dennett, 1991; Searle, 1992), progress has been made in recent years in cultivating pragmatics that help reduce the influence of bias, increase authenticity and evaluate reliability (Petitmengin, 2006; Olivares et al., 2015; Kordeš et al., 2019; Petitmengin et al., 2019a; Valenzuela-Moguillansky and Vásquez-Rosati, 2019). Following other scientific domains, data obtained through phenomenological inquiry is not taken at face value as infallible but examined, interpreted, analyzed for invariant structures and generalized in various ways. Eventually, the status of 1P accounts is not determined by their facticity, but evaluated through procedural standardization, potential replication of its findings and intersubjective validation with other first, second and third -person methods. As Varela suggests:

“The usual opposition of first-person vs. third-person accounts is misleading. It makes us forget that so-called third-person, objective accounts are done by a community of concrete people who are embodied in their social and natural world as much as first-person accounts […]. The line of separation — between rigor and lack of it — is not to be drawn between first and third person accounts, but determined rather by whether there is a clear methodological ground leading to a communal validation and shared knowledge” (Varela, 1996, p. 340).

It is this methodological ground which we aim to advance. As practitioners of integrated 1P and 3P research, we have gained some experience in the pragmatics of neurophenomenology. Thus, we are far from naively suggesting that 1P methods can be added into neuroscience without reflection, but rather hope to further illuminate some practical considerations that address inherent challenges.

**FIGURE 1:**
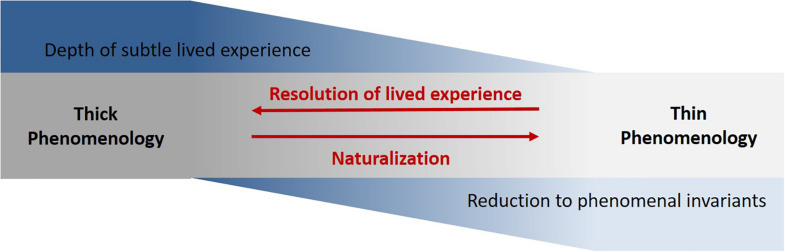
Tension and trade-off between phenomenological thickness and naturalization. The thick and thin phenomenology extremes form a continuum of the depth of 1P data acquisition.

Embracing the pragmatic spirit of the NRP, we suggest framing the diversity of possible forms of 1P data on a continuum of complexity (Figure 1). On one pole there is data obtained through in-depth phenomenological interview methods (Bitbol and Petitmengin, 2016a; Petitmengin et al., 2017, 2019b; Ollagnier-Beldame and Cazemajou, 2019) characterized by highly refined, detailed and dynamic accounts of singular subjective experiences (such as data gathered through micro-phenomenology). We call this thick phenomenology, to denote the high complexity of 1P data. On the other pole are thin methods (e.g., self-reports and questionnaires), acquiring data which provides information relevant to subjective experience, but limited due to its reductive nature (using pre-defined rigid phenomenal invariants) which also makes it prone to biases (Polkinghorne, 1989; Hurlburt and Heavey, 2015). We call it thin, to denote the low level of complexity of this kind of 1P data. In between these two poles, various methods can be placed, for example structured interviews and Descriptive Experience Sampling (DES, Hurlburt and Akhter, 2006)^2^.

The continuum directly relates to the possibility of naturalizing subjective experience, which in this specific respect refers to the prospect of integrating 1P account within an experimental neuroscientific setup^3^. At the thinner end of the continuum, data can be acquired rapidly, repeatedly and uniformly, facilitating intersubjective and cross-situational generalization. This is often relevant for its integration with neural measures because these typically require a larger number of sampled timepoints and individuals in order to yield reliable results. Furthermore, thin 1P data is easier to formalize and often quantifiable and thus more suitable for guidance of 3P data analysis, as well as easier accessible for scientific dialogue and cross-validation. Conversely, it is often limiting, unreliable and tainted by artifacts as it fails to address the multi-layered intricacy and dynamics of lived experience and to bracket assumptions and presuppositions of respondents (Hurlburt and Heavey, 2015; Bitbol and Petitmengin, 2016b).

Thicker methods of investigation result in much more refined accounts of experience, potentially amounting to more authentic descriptions of experience sensitive to its multi-dimensional and diachronic nature. Their skillful execution, analysis and formalization are thus far more cumbersome and require meticulous effort. They are, on the one hand, less accessible for generalization and quantification, but on the other hand, their high resolution may permit the bridging of experiential and neural microdynamics (as proposed by Petitmengin and Lachaux, 2013). Refined accounts of experience are also more receptive to novel insights which can guide future research. Overall, the naturalization of these methods through efficient integration within an experimental setup remains a challenging endeavor.

Rather than advocating the use of one method over the other, we suggest that this methodological trade-off is essential to the open-ended circulation envisioned by the NRP. As elaborated in the next section, we found it helpful in our own studies to alternate between different methods depending on factors regarding the available neuroscientific and phenomenological resources, as well as considering the current level of understanding of the studied phenomena. Such an integrative mixed-methods approach is useful in the triangulation of acquired data, and enjoys the advantage of using precise techniques that access specific and distinct dimensions of lived experience (further discussed in section “Building Bridges Between Phenomenology and Physiology – Mutual Constraints”).

### Cooperation With Meditators as Skilled Participants

As 1P data is fundamental for the NRP, scientific cooperation with “phenomenologically trained subjects” has been suggested to be useful (Thompson et al., 2005, p. 45). While such training is effortful and time-consuming, senior meditation practitioners have been proposed by several authors as pre-trained subjects suitable for such inquiry, based on the similarity between the epoché and certain meditative techniques (Varela et al., 1991; Varela and Shear, 1999; Depraz et al., 2003; Bitbol, 2019a; Depraz, 2019; Kordeš et al., 2019; Vörös, 2019). For example, Bitbol (2019a) argues that the common characterization of mindfulness, “namely as concentrating on the present moment and staying non-judgmental, captures a fundamental feature of the epochè: not only the suspension of elaborate judgments, but even before that, the suspension of the semantic function of both mental and verbal activities, that tends to expel us from our present.” (Ibid., p. 136). Similarly, Varela suggested that certain forms of meditation from the Buddhist tradition can be conceived of as epoché:

The exercise of samatha, best translated as mindfulness meditation practice, is based on an examination of the nature of our mind, (and hence of the origin of habitual patterns) by paying meticulous attention to every moment of appearance. In other words, using the activity of mind to go beyond mind, looking at the givenness of experience with a fresh, inquiring glance (Varela, 2000, p. 5).

In addition to the cultivation of the ability to suspend judgments and maintain a fresh conceptual-free perspective, other potential assets trained meditators bring to phenomenology are the ability to volitionally reproduce specific features of experience as cultivated in a given meditative practice (Lutz, 2002; Lutz et al., 2007; Desbordes and Negi, 2013), as well as the ability to stay with the experience being studied, i.e., reduce getting ‘lost in thought’ and mind-wandering as is typical in untrained subjects (Mrazek et al., 2013).

While a number of authors have claimed that the cultivation of mindfulness improves the quality of introspection, it has not yet been sufficiently empirically established, yet some supportive evidence is available. For example, meditative practice was shown to correlate with the predictive accuracy of self-reports regarding behavior (Abdoun et al., 2019), neuroanatomy (Fox et al., 2012) and peripheral physiology (Parkin et al., 2014). The latter was also confirmed in a recent meta-analysis indicating a small but statistically significant positive relationship between mindfulness and objective measures of body awareness (Treves et al., 2019). This issue is still a matter of debate due to the complexity of its assessment.

It is important to mention that alongside substantial benefits, there are also concerns and drawbacks of harnessing qualified meditators as “phenomenologically trained subjects.” First, it might be hard for some meditators to examine their experience beyond the elements prescribed by their practice school, or to let go of the conceptualizations of their respective traditions (Kordeš et al., 2019). Second, the goal of the observation is different: while Buddhist practices aim at alleviating human suffering, the central motivation of phenomenology is knowledge (Bitbol, 2019a). Thus, in the long-term it might be beneficial for science to establish its own paradigms of contemplative (phenomenological) training as envisioned by Varela.

With this in mind, cooperation with meditators has proved to be useful for the NRP not only for their alleged familiarity with the phenomenological attitude, but also for their enhanced sensitivity to subtle aspects of their experience (see Jo et al., 2014), as well as their capacity to volitionally control and stably maintain specific conscious (and neural) states (for some empirical evidence, see Garrison et al., 2013, 2015). This last ability increases the signal-to-noise ratio and renders these features scientifically tractable. It is the combination of these elements borne by meditative practice that supports the scientific exploration of subtle aspects of consciousness as exhibited in our own research and that of others (Berkovich-Ohana et al., 2013a; Dor-Ziderman et al., 2013, 2016; Zanesco et al., 2013; Jo et al., 2014; Ataria et al., 2015; Lutz et al., 2015; Abdoun et al., 2019).

### Building Bridges Between Phenomenology and Physiology – Mutual Constraints

The working hypothesis of neurophenomenology is that phenomenological accounts of the structure of experience and their counterparts in cognitive science relate to each other through reciprocal constraints. According to Varela (1996):

It is quite easy to see how scientific accounts illuminate mental experience, but the reciprocal direction, from experience toward science, is what is typically ignored. What do phenomenological accounts provide? At least two main aspects of the larger picture. First, without them the firsthand quality of experience vanishes, or it becomes a mysterious riddle. Second, structural accounts provide constraints on empirical observations (p. 343).

**FIGURE 2:**
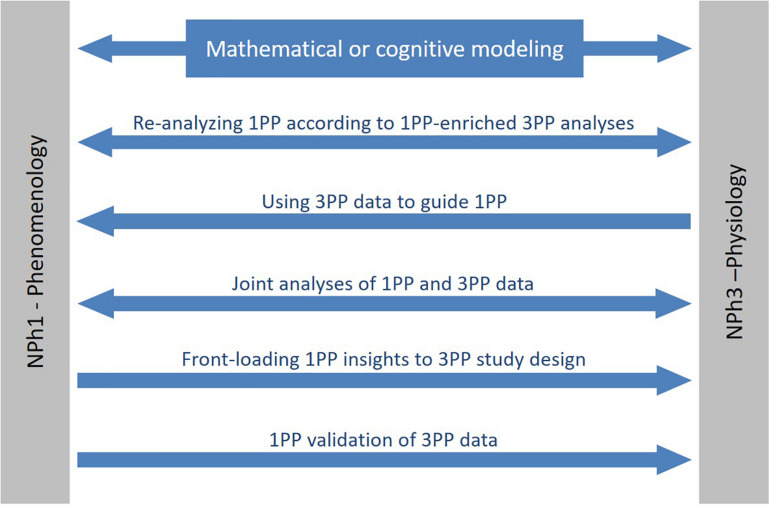
A typology of ways of bridging the irreducible domains of experience and physiology. NPh, neurophenomenology; 1PP, 3PP, first/third person perspective, respectively.

There are different ways to create bridges between the two irreducible phenomenal domains of experience and physiology. In this section, we offer a typology of previously proposed bridges, give an example to each of them, and schematically illustrate their directionality in Figure 2.

#### Bridge A: Front-Loading Phenomenological Insights Into Experimental Design

Gallagher (2003) and Gallagher and Sørensen (2006) suggested as a bridge between phenomenology and physiology the ‘front-loading’ of phenomenological insights onto experimental design. In other words, to design experiments informed by phenomenological insights – developed in independently conducted phenomenological analyses, or from previous neurophenomenological experiments. Such an approach was successfully implemented in a series of neuroimaging studies on ownership and agency (e.g., Chaminade and Decety, 2002; Farrer and Frith, 2002), which relied on previously generated phenomenological insights (Gallagher, 2000), rather than implementing 1P measures in the studies themselves (detailed in Gallagher and Sørensen, 2006). Additionally, the researcher’s direct access to his own lived experience inevitably influences the design and interpretation of the results. Rather than sweeping it under the carpet, such influences are here acknowledged, reflected upon and refined so that they could enhance the quality of the research^4^ (Jack and Roepstorff, 2002; Gallagher and Zahavi, 2008). In Figure 2, ‘front-loading’ phenomenological insights onto experimental design is illustrated as an arrow from 1P to 3P, signifying the use of 1P to design 3P studies.

#### Bridge B: Phenomenological Validation of Neurobiological Accounts

Varela (1996) proposed that disciplined 1P accounts ought to play be an integral element in the validation of a neurobiological proposal, i.e., that any attempt to scientifically explain mind and consciousness must directly relate to the nature of our lived experience (Varela, 1996, pp. 344–345). An adequate theoretical framework is thus needed to characterize neurophysiology in suitable terms that can also address the essential structure and dynamics of experience (further explored in bridge e). Such an approach proved highly productive in the important works of Petitmengin, Navarro, and Le Van Quyen concerning seizure anticipation (Le Van Quyen and Petitmengin, 2002; Petitmengin et al., 2006, 2007). Since 1975, researchers have used EEG analysis for the prediction of seizures, including a preictal state, which is detectable a few seconds before the actual seizure onset on EEG. The group’s EEG work showed that seizures do not arise suddenly, but as a transition from the interictal to the ictal state. It also showed that seizures do not correspond to deficient functioning of localized brain areas, but rather to deficient functioning of neural networks. However, the authors write, “the synchrony analysis does not tell us anything about the way this transition and this deficit are (or are not) felt by the patient. It indicates the structure of the cerebral activity, not the nature of the subjective experience that could correspond to it.” (Petitmengin et al., 2007, p. 750). Indeed, a phenomenological investigation showed that all nine investigated patients experienced auras (ictal phenomena), while six experienced prodromes (preictal phenomena). Studying the phenomenological dynamics showed that seizures are preceded by (often pre-reflective) symptoms. In Figure 2, phenomenological validation of neurobiological accounts is illustrated as an arrow from 1P to 3P, signifying the use of 1P to enhance insight into 3P.

#### Bridge C: Joint Analyses of 1P and 3P Person Data

Lutz (2002) emphasized joint analyses of 1P and 3P data, which actually means that phenomenal reports guide the analysis of the neuroscientific data^5^. The utility of this approach was illustrated in the seminal work of Lutz (2002) and Lutz et al. (2002), which received much discussion (Jack and Roepstorff, 2002; Gallagher, 2003; Bayne, 2004; Overgaard, 2004). In this study, subjects were trained to recognize stable categories of experience (‘phenomenal invariants’), which related to their state of ‘preparedness’ for the onset of simple visual stimuli presentation. Reports were then grouped into ‘phenomenological clusters.’ The results provide an outstanding example of the tailored use of 1P data, as the ‘phenomenological clusters’ were shown to reflect variability in neuronal response which otherwise would have been considered as noise. For example, only one cluster (when subjects reported “steady readiness” to the stimuli, in contrast to either “fragmented readiness” or “spontaneous un-readiness”) correlated with high gamma band EEG synchronization over frontal electrodes before stimulation. Importantly, EEG synchronization and behavioral measures of reaction time both correlated with the subjective reports. In Figure 2, joint analyses of 1P and 3P data is illustrated by a double-headed arrow connecting 1P and 3P, signifying the mutual use of both domains: grouping 3P for analysis according to 1P categories determined within the same experiment (or the other way around).

#### Bridge D: Using Physiological Data to Guide Investigation of Subjective Experience

A meaningful bridge could be constraining and guiding 1P data via the physiology itself. An illuminating demonstration of such an approach is the fMRI-neurofeedback study by Garrison et al. (2013) and Van Lutterveld and Brewer (2015). This study assessed how the 1P experience of meditation relates to neural activity in a core region of the default mode network – the posterior cingulate cortex. Activity in this region was measured and displayed on a screen in real-time, enabling participants to realize how their experience related to changes in the graph. The researchers then analyzed 1P data consisting of meditators’ accounts of their subjective experience during runs of a real time fMRI neurofeedback, and 3P data consisting of corresponding PCC activity during the same runs. The results showed that for meditators, subjective experiences corresponding with PCC deactivation related to “undistracted and effortless awareness,” while the subjective experiences of “distracted and effortful awareness” corresponded with PCC activation. In Figure 2, using 3P data to guide 1P is illustrated as an arrow from 3P to 1P, signifying the use of 3P to constrain and study 1P data.

#### Bridge E: Re-analyzing the 1P According to the 1P-Enriched 3P Analyses

Another way of creating meaningful bridges between 1P and 3P data is implementing iterative processes, i.e., re-analyzing the 1P according to the 1P-enriched 3P analyses (Lutz, 2002). This is actually the 3rd stage of the formal NRP as proposed by Varela, and hence represents a maturation of one’s project. To the best of our knowledge, the only work which implemented this approach is described by Petitmengin et al. (2007) as a “neuro-phenomenological circulation process.” In this work focusing on seizure anticipation (detailed in bridge b), the discovery of a new neuro-dynamic structure (the preictal neuro-electric desynchronization) permitted a refinement of the corresponding experiential dynamics (preictal phenomenological symptoms and therapeutic countermeasures). In Figure 2, re-analyzing the 1P according to the 1P-enriched 3P analyses is illustrated by a double-headed arrow connecting 1P and 3P, signifying the iterative dynamic process connecting the two irreducible phenomena.

#### Bridge F: Mathematical or Cognitive Modeling

The two irreducible domains have been suggested to be bridged by developing formal mathematical or cognitive models with variables that can refer to either phenomenal or neurophysiological states, an approach previously referred to as “generative passage” (Lutz, 2002). As a general approach to the study of consciousness, this notion has been gaining traction in contemporary theories of consciousness, notably the Integrated Information Theory (Oizumi et al., 2014; Tononi et al., 2016). Other promising theoretical developments build on the free energy principle (Friston, 2009; Friston et al., 2018), offering ways of specifying formal computational models of the autopoietic, embodied and enactive mind (Allen and Friston, 2018; Bruineberg et al., 2018; Kirchhoff, 2018; Ramstead et al., 2018). The concept of predictive processing is here transformed into “predictive engagement” (Gallagher and Allen, 2018), and proposals of how core predictive processing dynamics relate to (pre-reflective) aspects of experience have been put forth (e.g., Seth, 2015; Allen and Tsakiris, 2018; Fabry, 2019; Lutz et al., 2019), calling for rigorous neurophenomenological evaluation. An exceptional project, which puts into practice this approach, is the Projective Consciousness Model (PCM), a mathematical model of embodied consciousness, which is based on the hypothesis that the spatial field of consciousness is structured by a projective geometry and controlled by active inference processes (Rudrauf et al., 2017). While still under development, the PCM helps to account for aspects of subjective character including pre-reflective self consciousness, the 1P point of view, the sense of ownership, and social self consciousness (Williford et al., 2018), hence providing a mathematical model tying together phenomenological and neural levels of descriptions. In Figure 2, Mathematical or cognitive models are illustrated by a double-end inflated arrow connecting 1P and 3P, signifying the need for formal language to connect the two irreducible domains.

Let us close the first part of the paper by suggesting to cease looking for one meaningful bridge between neuronal activity and subjective experience, and rather aim for multiple and diverse feasible bridges. Accordingly, we believe that it is a mistake to think about the NRP as one experiment in which the researcher should choose one of the phenomenological attitudes (thin vs. thick). A fruitful dialogue between 1P and 3P is created by using different kinds of methods on the proposed continuum, at different developmental stages of the NRP, aiming at different insights – each of which can be re-integrated to inform other stages.

One can start a neurophenomenological investigation by implementing the bridge of front-loading (preliminary) phenomenal insights into the NRP study design (bridge a). While executing the study, the phenomenological thickness applied can be guided by various factors such as the number of subjects and available experimental resources, the quality and specificity of the studied phenomenon (in terms of availability and temporal dynamics), the adequacy of available questionnaires and other behavioral measures. Likewise, it is fruitful to use a variety of experimental technologies, as different technologies are useful for different bridges. For example, electrophysiological complexity measures can be suited for comparison with thick phenomenological data and phenomenal validation of neurobiological data (bridge b); specific cognitive tasks might be best suited for measuring underlying mechanisms of specific aspects of experience which can then be analyzed jointly with 1P data (bridge c); neurofeedback is best suited for using physiological data to guide subjective experience (bridge d); and mathematical modeling is highly suitable for creating cognitive models (bridge f). Importantly, bridge e requires a mature NRP, with iterative experiments, and is thus rarely implemented (but is demonstrated in or studies, as detailed subsequently).

In the second part of the paper, we will show how creating a variety of bridges improved our understanding of both the phenomenological side, as well as the neural side, of the embodied self phenomenon we were studying. Yet more importantly, none of these neural or phenomenological aspects by themselves could have led to our current understanding of the embodied, minimal self. The gained insight was a result of re-analyzing 1P data according to the 1P-enriched 3P analyses, representing the maturation of the NRP.

## Some Lessons From Our Journey With NRP: Studying Self-Dissolution

In this section, we lay out a series of studies, demonstrating how harnessing neurophenomenology can advance the study of self consciousness. This direction of inquiry goes back to Varela, Thompson and Rosch’s seminal work, The Embodied Mind:

We believe that mindful awareness practices can provide a natural bridge between cognitive science and human experience (phenomenology). Particularly impressive to us is the convergence that we have discovered among the main themes concerning the self and the relation between subject and object (Varela et al., 1991; The Embodied Mind, p. 33).

We start with a brief review of the field of self consciousness, including basic phenomenological conceptualizations of types of self consciousness, related cognitive and neural counterparts, and methods of scientific inquiry. This is meant to provide a broad context for our series of studies, highlighting their contribution to the readers who are less familiar with this field (while others can skip to the next section).

### Studying Self Consciousness

An increasing number of publications in philosophy, psychology and neuroscience investigate “self consciousness” – or the “sense of self,” referring here to subjects’ consciousness of themselves. The concept of self is highly ambiguous and includes various aspects, thus it may be best construed as a multidimensional construct including somatosensory, agentive, narrative and social components (Gallagher, 2000; Strawson, 2000; Gallagher, 2011, 2013), involving various brain regions (Christoff et al., 2011; Vogeley and Gallagher, 2011; Northoff et al., 2006). As part of a dialogue between philosophy of mind and cognitive neuroscience, a fruitful distinction has been made between two types of processes contributing to the sense of self: self-related and self-specific processing (Legrand and Ruby, 2009; Christoff et al., 2011). The first, self-related processing, attributes or evaluates stimuli with respect to one’s perceptual image or mental concept of oneself, giving rise to an enduring sense of identity (such as when contemplating one’s own personality, traits, memories or appearance). The second, self-specific processing, specifies the self as an embodied subjective knower and agent. Self-specific features are defined as being exclusive and non-contingent, meaning that they characterize oneself and no-one else, and that changing or losing them entails changing or losing the distinction between ‘self’ and ‘non-self.’ Thus, self-specific processes are considered more primal as they implement a functional self/non-self, or self-world distinction in perception, action, cognition, and emotion (Christoff et al., 2011; Seth, 2013). This distinction overlaps with previous differentiations in the literature, such as the ‘Me’ as opposed to the ‘I’ (James, 1890), ‘extended’ vs. ‘core’ self (Damasio, 1999), and ‘narrative’ vs. ‘minimal’ self, respectively (Gallagher, 2000; Gallagher and Zahavi, 2008).

Self-related processes have received the bulk of empirical attention, given that they can be easily manipulated in the lab through cognitive tasks. Neural activations during those tasks overlap strongly with the default-mode network (DMN, Raichle et al., 2001), a large-scale intrinsic network which is highly active at rest (as compared to externally focused goal-directed tasks) as well as during internally focused cognition including self-reflection, episodic memory, future planning, theory of mind, and personal moral reasoning (Gusnard et al., 2001; Raichle et al., 2001; Northoff et al., 2006; Spreng et al., 2009; Murphy et al., 2018).

Studies of self-specific processing and the minimal self, on the other hand, are less common. Of particular relevance are studies on the neural basis of the senses of agency and ownership (Ionta et al., 2011), the subjective experience of owning and being in control of one’s body and thoughts (De Vignemont, 2011; Ionta et al., 2011; Sperduti et al., 2011; Herbert and Pollatos, 2012), as well as the sense of being localized within one’s physical body (Blanke and Arzy, 2005; Tsakiris et al., 2008). Cleverly designed experimental setups have managed to create whole body illusions in virtual-reality environments (Alsmith and Longo, 2019), which have been used to directly investigate the experiential and neural implications of manipulating the self-body unity in terms of self-identity, self-location and 1st-person perspective (Kilteni et al., 2012; Serino et al., 2013; Guterstam et al., 2015). Regarding the underlying neuroanatomy, more than any other region, the above studies converge on the right temporoparietal junction and its neighboring regions, involved in multisensory integration and self-other distinction (Donaldson et al., 2015). Another approach to the study of self-specific processing is to investigate real-world cases in which senses of agency or body -ownership appear to be radically disrupted, including psychopathologies such as schizophrenia (Sass, 2013), post-trauma (Ataria, 2018), depersonalization disorder (Gerrans, 2018), and neurological disfunctions involving out-of-body-experience and autoscopy (Blanke and Arzy, 2005).

Importantly, however, an understanding of the neural processes underlying the fully fledged minimal self experience is still lacking. This is due to limitations of the above-mentioned approaches who study local alterations and disruptions of single features of self experience, such as the sense of agency (e.g., thought insertion), body ownership and self-location (e.g., full-body illusions). By contrast, there is emerging empirical evidence suggesting that some non-ordinary states of consciousness involve a more dramatic, global dissolution of the sense of self, and self-specific features in particular (Millière et al., 2018). This might be the case during dreamless sleep (Windt et al., 2016), drug-induced ego dissolution (Letheby and Gerrans, 2017; Millière, 2017) and deep meditative states (as shown by our neurophenomenological studies discussed below: Dor-Ziderman et al., 2013, 2016; Ataria et al., 2015). Of these, the only condition which can be non-chemically and volitionally manipulated in the lab is the deep meditative state. Thus, in addition to meditators’ general proficiency in experiential awareness (as discussed above), their specific meditative skills in generating states of global dissolution of self experience render them a uniquely apt cohort for the study.

Our approach to tackling this issue has been as a multidisciplinary team consisting of cognitive neuroscientists, empirical phenomenologists, and in collaboration with an expert meditator (who later became an integral part of the team), who demonstrated in the lab for the first time volitional malleability of the sense of boundaries (SB). Subsequently, we have been exploring meditation-induced neuro-oscillatory and experiential fingerprints of different modes of self consciousness, and ‘selfless’ states in particular, in highly adept meditators, via the neurophenomenological method (Berkovich-Ohana et al., 2013a; Dor-Ziderman et al., 2013, 2016; Ataria et al., 2015; Berkovich-Ohana, 2015). These studies are subsequently described in some detail.

### Previous Neurophenomenological Studies on Self-Dissolution

The studies outlined below demonstrate an evolving research effort, a blueprint, for taking a subject matter which is notoriously difficult to study, and of which very little is known – in terms of both its phenomenology as well as its neural mechanisms – and rendering it tractable to robust scientific investigation. In the present case, this process required three discrete stages to implement a full NRP. The first stage was a proof-of-concept feasibility study in which trained meditators produced deep contemplative states such as timelessness, spacelessness and selflessness under neuroscientific examination (Berkovich-Ohana et al., 2013a; Dor-Ziderman et al., 2013; Berkovich-Ohana, 2015). The second stage (Ataria, 2014; Ataria et al., 2015; Dor-Ziderman et al., 2016) consisted of a zooming-in process in which we: (1) gained more precision on the exact phenomenological construct under study by using thick phenomenological inquiry, (2) developed a suitable experimental setup and research environment, and (3) identified the necessary personnel – both expert meditators and researchers – for carrying out a more refined neurophenomenological study. The third stage, which is a project still underway (see section “A Mature and Comprehensive NRP on SB Dissolution”), lays out a robust, mature and comprehensive neurophenomenological research program centered on sense-of-boundaries dissolution and building on the insights gained from the first two stages. It is important to emphasize that exercising a gradual approach in this project was necessary, given how little was previously known regarding the experiential, as well as neural, dimensions of deep self-dissolution states. And furthermore, given that an established methodology for conducting such studies is still virtually non-existent.

#### Proof-of-Concept (Building the First Bridges)

The working basis for the study’s design was the assumption that long-term Buddhist-oriented mindfulness meditators would be able to produce and hold, volitionally and on demand, certain states pertaining to the self and its dissolution. This assumption was partly based on a preliminary pilot study, which reported two cases of altered states spontaneously occurring during meditation in two proficient practitioners (Berkovich-Ohana, 2015). These states of self dissolution are not uncommon occurrence for insight meditation practitioners and are considered the culmination of mindfulness-induced stages of consciousness. They are characterized by little-to-no conceptual thought and a disintegration of the ordinary subject-object intentional structure of consciousness, which is usually centered on the embodied sense of self. In the Buddhist tradition, these states are deemed highly valuable as they lead to important insights and realizations: “This comprehension of an object noticed, as being impermanent, painful, and without a self (impersonal) […], by means of simply noticing, without reflecting and reasoning, is called “knowledge by comprehension through direct experience” (Sayadaw, 1964, pp. 10–11; Shulman, 2014).” In this study, participants signaled immediately after the occurrence of such states in the lab, while electroencephalography (EEG) was continuously measured. After the meditation, the participants were asked to freely describe the signaled episodes. The preliminary results demonstrated a unique EEG pattern [an increase in global long-range gamma (25–45 Hz) synchronization] during the signaled states, compared to the background meditation state. Importantly, this preliminary case-study illustrated the feasibility of experiencing spontaneous deep meditative states of self-dissolution in the lab. The phenomenology employed was rather thin, due to the researcher’s lack of training in the phenomenological method, yet it allowed the creation of the very first bridge: front-loading 1P insights to 3P study design (bridge a). The neural analysis employed dynamic connectivity within ongoing EEG measurement, thus also enabling phenomenological validation of neurobiological accounts (bridge b).

The next study already recruited a larger cohort of experienced meditators for investigating a range of facets specific to the sense of self. In designing tasks for producing the desired phenomena, we relied on Gallagher’s (2000) influential conceptualization of self consciousness as “narrative self” (personal identity with temporal extension) and “minimal self” (momentary awareness rooted in bodily and multisensory processes, endowed with a sense of agency, ownership and 1st person perspective). Our aim was to map the patterns of neural activity underlying narrative and minimal states. However, by front-loading previous phenomenological insights, we also added what we called ‘selfless’ states, a present-moment awareness devoid of a subjective self core^6^. Rather than define it beforehand, the study’s aims were to both characterize this state phenomenologically, as well as to capture its underlying neural fingerprint. The experiment’s sample consisted of sixteen long-term meditators tasked with repeatedly producing and holding states pertaining to the narrative self, minimal self, as well as states devoid of the sense of self (Dor-Ziderman et al., 2013). Simultaneously, their brain activity was recorded by magnetoencephalography (MEG), a technology directly measuring the magnetic fields produced by the brain’s neurophysiology at a high resolution. It enables differentiating brain activity occurring at different frequency bands including fast brain rhythms, as well as reconstructing their cortical sources. Furthermore, the MEG is setup in a quiet, dark and heavy magnetically shielded room. It is non-invasive and there is no interference from the equipment during the experiment. These factors allow creating a relaxed and intimate environment suitable for the generation of deep meditative states. Each state was produced three times in succession, for 30 s.

As the study was exploratory, and we were not yet experienced with the NRP, we employed retrospective self-reports, as well as two different measures of 1P reports. One was extremely thin, to enable direct naturalization, and the second somewhat thicker:

(a) First, participants evaluated on a 1–3 scale their degree of success in producing each state. The purpose of these numerical reports was to exclude from neural analyses the subjectively non-successful selfless states (ratings of 1). This was done in the MEG immediately after producing each state. Retrospective reports regarding the meditators’ perceived (relative to past experiences) success and stability in performing the tasks (on a 1–10 scale, with 1 denoting “very low” and 10 denoting “very high”) were collected after the MEG session. Using a similar design, the participants also produced dissolution states in the time and space dimensions (reported in Berkovich-Ohana et al., 2013a). The finding that emerged from these self-report measures was that our participant population, i.e., Buddhist-oriented mindfulness practitioners, were more capable of successfully producing and stably maintaining dissolution states in the self dimension relative to the time and space dimensions (Berkovich-Ohana et al., 2013a; Dor-Ziderman et al., 2013). This finding is coherent with the emphasis placed in Buddhist practice on such experiences. It echoes Varela’s suggestion that long-term meditators are especially suitable subjects for studying self experience (Varela et al., 1991), and in particular its subtler pre-reflective aspects (Varela, 2000), within the NRP framework.
(b) Second, following the neural recordings, while still lying within the MEG and via an intercom, participants were asked to briefly describe their experience in the ‘selfless’ condition freely and in their own words, without reflection or judgment (Jack and Roepstorff, 2002; Schooler, 2002; Lutz and Thompson, 2003). The collected phenomenology was of medium degree on the thin-to-thick continuum due to the lack of skilled phenomenological investigators, as well as technical limitations of conducting interviews during MEG recordings. The short descriptions did, however, make their categorization and validation simpler. The ‘selfless’ phenomenological descriptions were analyzed and divided into three categories which were then validated by 12 independent judges. These categories indicated either (1) a quieting of experience, (2) an altered dream-like state, and (3) a state lacking sense of ownership or agency. Interestingly, but not surprisingly, the phenomenal categorization was found to be linked with the degree of meditative experience (such that the most experienced meditators were all in the third category). The three phenomenal invariants produced by this process were later used to contrast the third group with the other two, in order to underpin the neural signature of this radical phenomenological shift.

The experiment’s neural results indicated that different modes of self-processing involved dissociable frequency-dependent networks (Dor-Ziderman et al., 2013). Narrative, time-extended reflective self-related processing was marked by extensive frontal, and medial prefrontal gamma band (60–80 Hz) power, marking attenuation of default-mode activity, in line with fMRI (Whitfield-Gabrieli et al., 2011) and intracranical EEG studies (Nir et al., 2007). In contrast, minimal self processing was linked with beta-band (13–25 Hz) power in a more posterior network including medial (precuneus and posterior cingulate) and inferior parietal lobule (IPL) regions. Contrasting the last phenomenal category with the first two revealed a further right IPL beta-band power reduction, thus linking together phenomenology, meditation experience as well as a distinct neural signature.

To summarize, the neurophenomenological bridging principles we used in this study included: Front-loading 1P insights to the 3P study design – building on the participant’s ability to produce ‘selfless’ states on demand (bridge a); 1P validation of 3P accounts – by collecting phenomenology of the ‘selfless’ states (bridge b); and joint analyses of 1P and 3P data, by creating three *post hoc* phenomenal categories and using them to contrast the sub-groups and gain new insight which otherwise would not have been available (bridge c).

#### Zooming-In

The next steps involved thick phenomenology, zooming into the selfless experience with the aim of understanding the phenomenon in terms of both phenomenology and neurophysiology. This thickening of the phenomenological data collection and analysis was enabled by the close collaboration with Y. A., an expert in the phenomenological method.

We began with an in-depth phenomenological study in which 27 advanced mindfulness meditators were interviewed (Ataria, 2014). The goals of this study were:

(1) Mapping the subjective experience during meditation in general terms.
(2) Defining the ability of different meditators to describe their own experience during meditative states (in terms of depth and thickness).
(3) Identifying specific structures in the intentional arc that underwent changes during meditation.
(4) Identifying changes in the meditators’ sense of boundaries and sense of self.
(5) Exploring the sense of body ownership during meditation.
(6) Identifying meditators with the ability to volitionally replicate their experience.

At this stage, we were ready for a more detailed case study, examining both the phenomenology and the mediating neural substrate of a well-defined phenomenological construct which emerged from Ataria’s (2014) study, namely, the sense of self-world boundaries. At this point, building on insights from an intimate workshop on neurophenomenology^7^ which highlighted the necessity to engage in a real and deep dialogue with experts well familiarized with deep contemplative states, we started working in full cooperation with a highly qualified meditator. We were lucky to be connected with a uniquely suited meditator, S.F., a former scientist and well-known meditation teacher with over 40 years and tens of thousands of hours of meditation experience. S.F.’s qualifications were based on both phenomenological as well as neural considerations. Based on phenomenological descriptions provided in previous studies (Berkovich-Ohana et al., 2013a; Dor-Ziderman et al., 2013; Ataria, 2014, 2020) and summarized in Ataria et al. (2015), S.F. stood out as a uniquely apt candidate, able to produce deep meditative states on demand, in a differentiated, replicable and stable manner. In addition, S.F. could describe his experience in clear and precise language, as it was unfolding. Furthermore, S.F.’s neurophysiological data from previous MEG (Berkovich-Ohana et al., 2013a; Dor-Ziderman et al., 2013), EEG (Berkovich-Ohana et al., 2012, 2013b), and fMRI (Berkovich-Ohana et al., 2016a, b) studies on self consciousness, indicated clear and strong effects, which reflected group-level processes (see Ataria et al., 2015). In other words, it was likely that S.F. would be able to produce the required states, describe them in clarity and detail, and that the corresponding neural data would be differentiable between-conditions and generalizable (not idiosyncratic).

The decision to focus on the sense of boundaries (SB) was a result of a discussion between the researchers (Y.A., A. B-O., and S.F.), regarding what would be the best phenomenological dimension that S.F. could alter by demand in the lab. S.F. identified the SB as a phenomenal continuum he could traverse very skillfully, moving along it in a precise way, and stopping in several reliable and differentiated states. SB dissolution is a central goal and skill of Buddhist meditation and has profound implications to the study of self. Albeit a relatively novel research field, there are indications regarding its prevalence among long-term meditators (Lindahl et al., 2017). We decided to focus, for the sake of simplicity, only on 3 highly differentiated phenomenal states ranging from a normal sense of boundaries (SB1) to a state in which the SB was beginning to dissolve (SB2) to a state in which the SB was completely absent (SB3).

The case-study design inherently included the three bridging principles practiced in our previous study: (1) Front-loading 1P insights to 3P study design – building on a series of preliminary phenomenological interviews and discussions with the practitioner S.F., as well as our prior studies; (2) 1P validation of 3P accounts – by collecting phenomenology of all three SB states; and (3) Joint analyses of 1P and 3P data, by creating ad-hoc three phenomenal categories and using them as ‘cognitive tasks’ to guide the MEG data acquisition and analysis.

The study was set up such that the phenomenological interview was conducted in similar conditions to the subsequent MEG experiment. SF generated the default, dissolving, and disappearing states, SB1, SB2, and SB3, for 1 min each, in succession, for four cycles, while his brain activity was recorded by MEG. We employed thick phenomenology – lengthy in-depth interviews conducted and analyzed by an expert empirical phenomenologist (for more details see Ataria et al., 2015). The thick phenomenology significantly advanced our understanding of the lived experience underlying the ‘no-self’ state in a number of important respects. While in the previous study care was taken to elicit 1P descriptions of the selfless state, their phenomenology was thin with little detail, richness and specificity. Additionally, the thin methodology was not conducive of the practice of ‘bracketing’ and thus a layer of Buddhist conceptualization (terms such as ‘emptiness,’ ‘liberation,’ and ‘witnessing’) could still be detected in the descriptions. Third, despite the emphasis placed by Buddhist traditions on ‘no-self’ experiences as a key to liberation, hardly any phenomenological documentation of such experiences exists due to taboos around discussing such experiences with anyone but one’s teacher. The thick analysis revealed SB dissolution experience to be a graded phenomenon, manifesting as nine experiential categories such as diminished or absent sense of agency, ownership, location, egocentric perspective and internal vs. external (Ataria et al., 2015).

In accordance with the diminishing quality of the phenomenal categories, at the level of the brain these changes were mediated only by beta band reductions, with no increases in activity. These beta reductions were localized to bilateral medial and lateral parietal regions (Dor-Ziderman et al., 2016), in particular the right temporal-parietal junction (TPJ, which includes the IPL), in line with our previous study (Dor-Ziderman et al., 2013). These results were coherent with the existing literature as the TPJ, more than any other region, has been shown to mediate the experiential unity of self and body, relying on multisensory integration and contributing to a sense of ownership, agency and self location (Tsakiris et al., 2008; Ionta et al., 2011).

While highly interesting and informative, these results still raised two important concerns. The first regarded the uniqueness of SF in terms of neural patterns. Could these results be generalized to a large population? The second concern regarded the gap between the high complexity of the phenomenology as compared to the neural findings. In other words, while the phenomenology produced nine phenomenal categories, the underlying neural mechanism was linked in previous literature to only some of these categories, so we were unable to discriminate which of the involved SB dissolution phenomenological categories was driving the neural results.

The need to further develop an understanding of the specification of the neural activity related to these experiential changes necessitated a novel study to be designed (the current team project, see section “A Mature and Comprehensive NRP on SB Dissolution”). However, we were already in position to address the first issue based on our previously collected data. By doing so, we implemented for the first time the advanced bridging principle of re-analyzing 1P based on 1P-enriched 3P data (bridge d). Specifically, our better grasp on the SB phenomenology shed new light on group data from our previous study (Dor-Ziderman et al., 2013), and it became apparent that they too demonstrated a form of SB dissolution during the selfless state. Hence, armed with the 1P-enriched 3P data, we could go back to their data and study the exact frequency and regions of interest. As a result, the case study’s neural results were partially validated (right hemisphere only) in a larger group (*n* = 10), and their specificity to the domain of self (not manifesting in control states focusing on the time and space domains) was demonstrated (Dor-Ziderman et al., 2016).

To summarize, this series of studies enabled us to show that it was possible to create, and validate, multiple advanced bridges between thick phenomenology and neuronal activity. The insight gained from both the thick phenomenology, as well as the MEG results, led our team to the third stage, a robust, mature and comprehensive NRP centered on volitional^8^ SB dissolution^9^. This project is still underway and is subsequently briefly described.

### A Mature and Comprehensive NRP on SB Dissolution

The earlier studies provided phenomenological support for the notion that meditators can profoundly alter their SB in meditation, and the neurophysiological results showed that these alterations were mediated by neural processes linked with embodied self experience in other streams of research (Blanke and Metzinger, 2009). These advancements set the stage for a mature, larger ongoing study aiming to take our NRP one step further. The main aims of this project are (1) specifying underlying neurocognitive models explaining the experiential categories and neural results, and (2) exploring individual differences (neural and phenomenological) borne of the mapping of SB dissolution into phenomenological clusters.

#### Specifying Underlying Neurocognitive Mechanisms

We aim to specify a cognitive model that is coherent with both the phenomenological and physiological levels of description. Neurophysiologically, this requires the specification of measurable neural parameters relating to key processes within such a model. In the current study, we approach this goal by assessing three candidate neural processes arising from previous empirical research and theoretical reasoning:

(1) The first potential mechanism is the integration of interoceptive signals, previously suggested to give rise to an affectively colored sense of the embodied self (Seth, 2013; Seth and Tsakiris, 2018). This mechanism is indexed in our study using the heartbeat evoked potential, a neurophysiological brain response time-locked to the heartbeat, shown to reflect interoceptive processing of cardiac signals (Schandry and Montoya, 1996; Gray et al., 2007). This measure is recorded and computed during various levels of SB production.
(2) A second potential mechanism is the integration of (motor) efference copies with their actual sensory consequences (re-afferences) (Christoff et al., 2011). Similar and more specific suggestions have been made for the sense of agency (Gallagher, 2000; Haggard and Chambon, 2012), where the suppression of neural responses to self-caused events (as compared to externally caused events) is regarded as the result of efferent/re-afferent integration (i.e., cancelation of sensory changes predicted through efference copies) (Baess et al., 2011). This effect has been shown to correlate with the subjective experience of agency (Gentsch and Schütz-Bosbach, 2011; Timm et al., 2016). This mechanism is indexed in our study using action-induced sensory suppression in a simple task involving button pressing and auditory events, in combination with meditative modulation of the SB.
(3) Finally, a third candidate mechanism is based on multisensory integration accounts of bodily self consciousness (Blanke, 2012; Blanke et al., 2015), which among other methods, has been investigated in peripersonal space paradigms (Blanke et al., 2015; Salomon et al., 2017). We test for peripersonal space modulations during SB dissolution experiences by adapting a previously used neurophysiological multisensory stimulation paradigm (Bernasconi et al., 2018).

By measuring these candidate processes and mapping participants onto related phenomenological dimensions, we aim to establish robust empirical bridges intertwining these two domains.

#### Individual Differences in Phenomenological Mapping

We aim to explore whether individual differences in phenomenal characteristics map onto different mechanisms. For this purpose, we have recruited a large sample of 50 meditators with a large variance in meditation expertise (115–24,837 h). For creating a mutual phenomenological language regarding the concept of the “sense of boundaries,” as well as for increasing the meditators’ prospects of successfully producing in the lab clear and stable dissolution experiences, we implemented a 3-week specially tailored meditative training developed and guided by S.F. Following the training, participants were invited to the lab and underwent a varied array of neural and behavioral tasks, phenomenological interviews, self-rating as well as questionnaires. This project is, to our knowledge, the most comprehensive examination to date of the nature of human self-boundaries experience and its neural, behavioral and experiential manifestations.

While the specific training helped focusing participants specifically on this aspect of their meditative practice, the large cohort of meditators entails an unavoidable heterogeneity and richness in participants’ meditative experiences of SB alteration. Therefore, an in-depth phenomenological investigation is necessary to make sense of the experiential diversity in a systematic manner. The phenomenological investigation is being conducted using a mixed-methods approach featuring epoch-based self-ratings of stability and depth of the meditative experience (thin phenomenology), follow-up questionnaires and semi-structured qualitative interviews (thick phenomenology). The interviews are conducted based on the open-ended, iterative questioning principles of the Micro-phenomenology method, producing detailed in-depth descriptions of the lived experience of the study participants (Petitmengin, 2006).

Integrating these methods will allow specifying and differentiating the various types of meditative experiences associated with SB alteration, and address the different phenomenological features described by the participants. The interview analysis will capture such diversity by assessing these experiences according to fundamental facets of self-experience such as the sense of location, sense of agency, attentional disposition and affective state. These facets were defined in a top-down fashion, partially based on the previous characterization of SB dissolution (described above), and partially in order to provide subjective parameters corresponding to candidate processes available in the literature (described in the previous section). Within each of these phenomenological categories, there are emerging patterns of variability that characterize and distinguish distinct types of SB dissolution. An additional category that emerged from the preparatory training and preliminary interview analysis is the type of technique (or inner gestures) involved in the dissolution process. Although trained together, participants performed a diverse set of meditative techniques which accordingly produced several distinct variations of the state of SB dissolution. We thus hope that by relying on a larger and diverse sample we can enlighten specifics and commonalities in the enactive dynamics of SB constitution and dissolution. In addition to these thicker aspects of SB dissolution phenomenology, repeated self-ratings throughout the experiments provide temporal tracking of fluctuation in the degree of depth and stability of meditation for each subject.

This full-blown NRP project attempts to implement all the proposed bridges: (1) Front-loading 1P insights to 3P study design – building on the fine-grained phenomenological analysis from the previous case study; (2) 1P validation of 3P accounts – by collecting phenomenology of reduced and enhanced SB states; and (3) Joint analyses of 1P and 3P data, by creating *post hoc* phenomenal categories and using them to guide the MEG data analysis; (4) Using 3P data to define 1P – by using cognitive tasks which engage different aspects of the embodied self (e.g., peripersonal space), we harness the accumulated 3P knowledge related to these tasks to constrain the phenomenology; (5) Using 1P-enriched 3P data to reanalyze 1P – by utilizing the previously found neural markers (in the case study and the small, described in Dor-Ziderman et al., 2016) to refine and build a semi-structured interview focusing on agency, ownership and self location; and (6) Cognitive modeling – we hope to be able to elucidate specific cognitive mechanisms underlying SB flexibility which might, eventually, be integrated into a comprehensive model of embodied self experience.

## Discussion

In this paper, we outlined the NRP’s requirements, explored its inherent tensions and suggested a typology of bridging principles, constraining the two irreducible domains of 1P and 3P. We then demonstrated the usage of these bridges by describing the unfolding of a series of studies investigating the experience of boundaries of the self, both phenomenologically and neurally. In both realms, the accumulated knowledge was quite limited due to taboos on publicly sharing such 1P accounts and experiences, the difficulty of manifesting such states volitionally, on demand and under experimental settings, and the lack of suitable cognitive modeling to guide the study. Hence, it is not only that exploring the subtle aspects of self consciousness supports and validates the NRP, but also the reverse, that the NRP is needed to handle such a subtle, profound, fascinating and challenging research topic as conscious experience.

We hope to have been able to demonstrate the potential of the NRP to inform the science of consciousness. We further hope that this account suitably narrates both the challenges and the creative solutions which were needed to be implemented along the way, in order to push this project forward. The guiding intuitions were always in the spirit originally put forth by Varela’s NRP of harnessing a pragmatic and flexible stance along the way, collaborating with well-trained participants, and above all, the indispensable need to treat human experience with equal importance as physiological data.

We consider this ongoing circulation between the two realms of physiology and human experience as an act of art, a deep listening, an improvisational dance, which slowly develops into a skillful scientific dialogue. It is not meant for those who use science as a battle to win, or as growing a tail to wag. What is required is passionate teamwork, a willingness to be re-enchanted with the realm of the living and to tackle the mystery of human consciousness from as many angles as possible while practicing pragmatism, flexibility and humility along the way.

## Author Contributions

AB-O: conceptualization, resources, supervision, investigation, and writing – original draft. YD-Z: methodology, software, data curation, formal analysis, investigation, and writing – original draft. F-MT, YS, and ON: investigation and writing – original draft. SF: writing – review and editing. YA: conceptualization, supervision, investigation, and writing – original draft. All authors contributed to the article and approved the submitted version.

## Conflict of Interest

The authors declare that the research was conducted in the absence of any commercial or financial relationships that could be construed as a potential conflict of interest.

## Footnotes

1 This therefore is a method enabling the gathering of “first person” data, i.e., data that express the viewpoint of the subject himself, in the grammatical form “I…”. But as these data have been gathered through another person (a “You”), it has been dubbed a “second person” method (Petitmengin, 2006, p. 231).

2 In DES, pre-trained participants are ‘beeped’ randomly, thus ordered to record (verbally or in writing) their inner experience in that particular moment. DES would be placed in between the two poles, as the resolution of the lived experience obtained in this method is lower, in part, in order to minimize bias of reconstruction. Importantly, it is used to inspect random, rather than specifically meaningful instants of experience.

3 The issue of naturalization of phenomenology clearly exceeds the specific context in which it is discussed here. It is further explored through the typology of bridges provided in Section “Building Bridges Between Phenomenology and Physiology – Mutual Constraints.” For theoretical accounts of naturalization of phenomenology, see Petitot et al. (1999), De Preester (2002), Overgaard (2004), Zahavi (2004), Gallagher (2012).

4 This is especially crucial in the skillful execution of 2P research (Petitmengin et al., 2019a, b).

5 This is also an example of front-loading, as Lutz et al. (2002) did pilot runs to identify the categories, which were then incorporated into the design.

6 The word ‘selfless’ is used to denote the state of lack of minimal self features, but some authors define it as a minimalist notion of self consciousness which implies its essential reflexivity or first-personal givenness (Zahavi, 2011).

7 A round table on neurophenomenology (December 2011) with scholars from different fields as well as meditators organized by AB-O and YA at the Inter-Disciplinary Center (IDC), Herzliya, Israel.

8 It is important to clarify that ‘volition’ as expressed here is more about a radical letting go than about control; more like a state that can be intentionally cultivated, or recognized.

9 Volitional SB dissolution cannot be equated with full realization of no-self; rather, it should be viewed as an experiential manifestation of a degree of no-self/emptiness insight.
