# Supplementary material for: The Hitchhiker’s Guide to Neurophenomenology – The Case of Studying Self Boundaries With Meditators
Source: Front Psychol. 2020 Jul 21;11:1680. doi: 10.3389/fpsyg.2020.01680 (PMC7385412; doi:10.3389/fpsyg.2020.01680)
Supplement: Supplementary file 1 [file Table_1.DOCX]

***Appendix 1: Historical roots***

In the early 20^th^ century, Edmund Husserl (1900/1970), later to become known as the father of the phenomenological movement, issued a call to contemporary psychologists urging them to treat subjective experience more seriously. Husserl (1965) took note of the existence of reciprocal relations between *empirical psychology* and *phenomenological psychology*, both focused on understanding the human mind, albeit from very different perspectives.

Before continuing, it is important not to confuse *phenomenological psychology* with *transcendental phenomenology*, two very different phenomenological approaches to consciousness Husserl distinguishes between. Zahavi (2004) emphasizes that while both approaches deal with consciousness, they do it from what seem to be, at least for Husserl, very different perspectives:

For Husserl, the task of *phenomenological psychology* is to investigate intentional consciousness in a non-reductive manner, that is, in a manner that respects its peculiarity and distinctive features. *Phenomenological psychology* is consequently a form of descriptive, eidetic, and intentional psychology which takes the first-person perspective seriously, but which—in contrast to *transcendental phenomenology*, that is, the true philosophical phenomenology—remains within the natural attitude… *transcendental phenomenology* is a much more ambitious global enterprise. It is interested in the constitutive dimension of subjectivity, that is, it is interested in an investigation of consciousness in so far as consciousness is taken to be a condition of possibility for meaning, truth, validity, and appearance...The most important point for the current discussion is that "*transcendental phenomenology* might be inherently opposed to the project of naturalization, the fact of the matter seems different when it comes to phenomenological psychology. (Zahavi, 2004, p. 339).

With this in mind it is quite clear why, at least from Husserlian perspective, the dialogue between phenomenology and psychology is between phenomenological *psychology* and *empirical psychology*. Yet even in this case there seems to be an inherent tension. Broadly speaking, *empirical psychology* is influenced by methodologies used in the natural sciences. Such methodologies aim to establish a strictly objective perspective, observing phenomena from an external third-person perspective. By contrast, *phenomenological psychology* embraces the fact that experiences are experienced in some way and from within, that is, from a first-person perspective. Nevertheless, Husserl argues in favor of such dialogue – at least under some constraints:

With this we meet a *science* of whose extraordinary extent our contemporaries have as yet no concept; a science, it is true, of consciousness that is still not psychology; a phenomenology of consciousness as opposed to a natural science about consciousness. But since there will be no question here of an accidental equivocation, it is to be expected beforehand that phenomenology and psychology must stand in close relationship to each other, since both are concerned with consciousness, even though in a different way, according to a different "orientation." This we may express by saying that psychology is concerned with “empirical consciousness,” with consciousness from the empirical point of view, as an empirical being in the ensemble of nature, whereas phenomenology is concerned with “pure” consciousness, *i.e.*, consciousness from the phenomenological point of view. (Husserl, 1965, pp. 173-4)

In order to truly grasp the phenomenological project (and the neuro-phenomenological project by consequence) one needs a clear understanding of what Husserl means by first-person perspective. In terms of *phenomenological psychology* (once again, not to be confused with *transcendental phenomenology*), the goal of the phenomenological method is to describe lived experience (Husserl, 1900/1970; 1936/1970), or in other words, how we are thrown into the world pre-reflectively. This requires what has been called by Husserl Epoché or “bracketing" – meaning that we must suspend judgment about the "natural world" and withdraw old beliefs, conceptions, and opinions. The phenomenological approach argues that we cannot observe consciousness just as we would observe any other ordinary research object. Indeed, in the context of empirical psychology, introspection does not relate to subjective experience as a unique phenomenon; rather, it is an object like any other in nature (Jennings, 1986). While Husserl did not oppose empirical psychology (natural psychology or cognitive science),[1] or even psychological introspection *per-se*, he suggested that when studying consciousness, in particular the intentional structure of consciousness, psychological introspection is not useful – precisely because one cannot examine one’s own intentional structure as an object:

The whole thing, however, depends on one’s seeing and making entirely one’s own the truth that just as immediately as one can hear a sound, so one can intuit an "essence" – the essence "sound," the essence “appearance of thing,” the essence "apparition,” the essence "pictorial representation,” the essence "judgment" or "will,” etc. – and in the intuition one can make an essential judgment. On the other hand, however, it depends on one’s protecting himself from the Humean confusion and accordingly not confounding phenomenological intuition with "introspection,” with interior experience – in short, with acts that posit not essences but individual details corresponding to them. (Husserl, 1965, p. 83)

Husserl's Epoché can allow one entrance to the basic experience of being in the world, also referred to as pre-reflective self-consciousness:

pre-reflective self-consciousness is pre-reflective in the sense that (1) it is an awareness we have before we do any reflecting on our experience; (2) it is an implicit and first-order awareness rather than an explicit or higher-order form of self-consciousness… In contrast to pre-reflective self-consciousness, which delivers an implicit sense of self at an experiential or phenomenal level, reflective self-consciousness is an explicit, conceptual, and objectifying awareness that takes a lower-order consciousness as its attentional theme. I am able at any time to attend directly to the cognitive experience itself, turning my experience itself into the object of my consideration (Gallagher and Zahavi, 2008).

Thus, it is easier to understand why the common methods used in empirical psychology do not allow grasping this initial experience. Essentially, however, Husserl did not argue that objective measurements should be omitted from the study of consciousness. Rather, he suggested improving our understanding of subjective experience by combining the two approaches to the study of consciousness – phenomenological psychology and empirical psychology.

It is worth mentioning that this issue remains, at best, problematic since in his writing Husserl himself sometimes presented different perspectives on this issue:

Even Husserl himself, during the whole of his philosophical development, did not find it easy to determine once and for all his attitude toward psychology, and to define the exact function which he assigned to it within the framework of his changing conception of phenomenology. In any case, he kept stressing the particularly close connection between the two and asserted that his phenomenology was relevant to psychology and could be applied to it after an appropriate change of attitude (Spiegelberg, 1960, p. 149).

[1] "My criticism of psychological method did not at all deny the value of modern psychology, did not at all disparage the experimental work done by eminent men. Rather it laid bare certain, in the literal sense, radical defects of method upon the removal of which, in my opinion, must depend an elevation of psychology to a higher scientific level and an extraordinary amplification of its field of work" (Husserl, 1913/1982, p. XVIII)

**Bibliography**

Husserl, E. (1900/1970). *Logical Investigations.* London: Routledge and Kegan Paul.

Husserl, E. (1913/1982). *Ideas Pertaining to a Pure Phenomenology and to a Phenomenological Philosophy – First Book: General Introduction to a Pure Phenomenology* (Vol. 2). (F. Kersten, Trans.) The Hague: Martinus Nijhoff.

Husserl, E. (1936/1970). *The crisis of European sciences and transcendental phenomenology:‎ and introduction to phenomenological philosophy.* (D. Carr, Trans.) Evanston: Northwestern University Press.

Husserl, E. (1965). *Phenomenology and the Crisis of Philosophy.* (Q. Lauer, Trans.) New York: Harper and Row.

Jennings, J. L. (1986). Husserl Revisited. *American Psychologist, 41*(11), 1231-1240. doi:10.1037/0003-066X.41.11.1231

Spiegelberg, H. (1960). *Tbe Phenomenological Movement* (Vol. 1). The Hague: Martinus Nijhoff.

Zahavi, D. (2004). Phenomenology and the project of naturalization. *Phenomenology and the Cognitive Sciences, 3*, 331-347.

Zahavi, D., & Gallagher, S. (2008). The phenomenological mind. *London: Routhledge*, *244*.‏
